# Children’s Exposure to Secondhand Smoke during Ramadan in Jakarta, Indonesia

**DOI:** 10.3390/ijerph13100952

**Published:** 2016-09-26

**Authors:** Nurul (Nadia) H.W. Luntungan, M. Justin Byron, Melbourne F. Hovell, Laura J. Rosen, Annisa Anggraeni, Vaughan W. Rees

**Affiliations:** 1 Center for Indonesia’s Strategic Development Initiatives, Jakarta 10350, Indonesia; Nurul.Luntungan@gmail.com; 2 Lineberger Comprehensive Cancer Center, University of North Carolina at Chapel Hill, Chapel Hill, NC 27599, USA; jbyron@unc.edu; 3 Center for Behavioral Epidemiology and Community Health, San Diego State University, San Diego, CA 92182, USA; mhovell@cbeachsdsu.org; 4 Department of Health Promotion, School of Public Health, Sackler Faculty of Medicine, Tel Aviv University, POB 39040, Ramat Aviv 69978, Israel; 5 School of Public Health, Universitas Indonesia, Depok, West Java 16424, Indonesia; Annisa.Anggraenis@gmail.com; 6 Center for Global Tobacco Control, Department of Social & Behavioral Sciences, Harvard T.H. Chan School of Public Health, Boston, MA 02115, USA; vrees@hsph.harvard.edu

**Keywords:** secondhand smoke, tobacco, children, policy, Indonesia, behavioral ecological model

## Abstract

Secondhand smoke exposure (SHS) causes a disproportionate health burden for children, yet existing smoke-free laws are often poorly enforced. We monitored air quality while observing children and adult nonsmokers present in public venues during Ramadan, a period of Muslim religious observance marked by family and social gatherings, in Jakarta, Indonesia. A repeated-measures design was used to assess indoor air quality during and after Ramadan in 43 restaurants and in five smoke-free control venues. Fine particulate matter of 2.5 microns or less (PM_2.5_) was sampled. The average number of children and active smokers present in each venue was also observed. PM_2.5_ levels were significantly higher during Ramadan (mean 86.5 µg/m^3^) compared with post-Ramadan (mean 63.2 µg/m^3^) in smoking venues (*p* = 0.015). During Ramadan, there were more active smokers (*p* = 0.012) and children (*p* = 0.051) observed in venues where smoking occurred, compared with the same venues post-Ramadan. Poor enforcement of the smoke-free law in Jakarta has failed to protect children from SHS exposure in public venues during Ramadan. Collaboration between the government, NGOs (such as the Indonesian Cancer Foundation (YKI) and the Smoking Control Foundation (LM3)), religious leaders, and venue owners and managers must be developed to ensure that the comprehensive smoking bans apply to all venues, and that smoke-free laws are enforced.

## 1. Introduction

Secondhand smoke (SHS) exposure is the third greatest preventable cause of non-communicable death and disease globally, and accounts for the deaths hundreds of thousands of non-smokers annually [1,2]. Children are at high disease risk because of their less-well-developed immune and respiratory systems [3]. Comprehensive smoke-free laws, which ban all smoking in indoor, public areas, are an important means of preventing SHS exposure [2] and Article 8 of the Framework Convention on Tobacco Control (FCTC) requires parties to the convention to implement such policies [4,5]. This has encouraged increasing global adoption of smoke-free legislation, although adoption and enforcement lags in low- and middle-income countries, where a greater burden of SHS exposure falls on women and children [6]. Internationally, smoke-free laws have led to decreases in SHS exposure and smoking behavior, and to the prevention of adverse health outcomes, including among children [7].

In Indonesia, at least 85% of adults are regularly exposed to SHS in restaurants [8]. Among youth, the most common sources of SHS exposure are public places and the home [6,9]. Despite not being a signatory to the FCTC, some important smoke-free policies have been implemented in Indonesia [10]. In 2012, Indonesia’s central government legislated indoor smoke-free areas [10], although enforcement of smoke-free laws is carried out by regional governments [11]. Jakarta, the capital city of Indonesia, has a comprehensive ban with no exemptions for open air venues [11]. However, compliance with the smoke-free law remains a problem in Jakarta (the average compliance rate of 37% in early 2014 decreased to 20% in early 2015) [12]. Poor enforcement may be a significant factor in the continued high SHS exposure among Indonesian children: some 38.2 million, or 60% of children 15 years old and under are exposed to SHS, mostly in enclosed public places [9]. Likewise, a high prevalence of smoking, together with pro-social norms for tobacco use that reflect a permissive attitude toward tobacco use even among families, may be a significant factor in children’s SHS exposure in Indonesia [13]. Indonesia has the largest Muslim population in the world, and religious customs have an important potential role in shaping smoking behavior. During the holy month of Ramadan, many Muslims fast from sunrise to sunset. The first meal after sunset each night is *iftar*, a celebratory meal in which families, including children, often gather in public venues to break the day-long fast. This results in potentially high exposure to SHS in indoor venues at a time when many children are present. In 2010, Muhammadiyah, a leading Indonesian Muslim organization, declared indoor smoking *haram* (forbidden) for its members because it is contrary to Islamic laws [11]. The wide observance of Ramadan among the Indonesian public may provide an opportunity to communicate indoor SHS levels and the need for increased efforts to enforce Indonesia’s smoke-free laws.

Air quality monitoring has been widely used to document the contribution of smoking to indoor air pollution [14,15,16]. Tobacco smoking releases large quantities of particulate matter of ≤2.5 microns (PM_2.5_) that provides a valid marker for SHS [15]. The World Health Organization (WHO) recommends a maximum mean of 10 μg/m^3^ for annual exposure to ambient PM_2.5_ pollution [17]. The results of air quality monitoring are commonly used to educate governments, community organizations, and civil society about the benefit and importance of implementing and enforcing comprehensive smoke-free laws [4,5,14]. Recently, SHS monitoring in Jordanian restaurants found that PM_2.5_ levels during Ramadan were higher after *iftar* than when measured in the same venues during an earlier time of the day [18]. Just as local-level data collected globally have been used to promote smoke-free policies and support implementation and enforcement efforts, data on SHS emission levels collected during Ramadan might influence regulators as well as religious leaders to support the enforcement of current smoke-free laws [19]. These efforts may help to protect the health of Indonesian families and children, as they gather to celebrate a popular religious custom, as well as at all times of the year. Thus, Ramadan provides an opportunity to demonstrate the impact of poor compliance on SHS exposure among children [18].

Despite existing research that has documented a high prevalence of SHS among children in Indonesia, there is little information on the social and cultural factors that contribute to SHS among children, including compliance with local smoke-free laws. The purpose of this study was to document indoor air pollution levels measured while children and adult nonsmokers were present, in a sample of hospitality venues in Jakarta during Ramadan, and compare outcomes in the same venues sampled post-Ramadan. 

## 2. Materials and Methods

### 2.1. Design 

A repeated measures design was used to measure indoor air quality in 43 restaurants in Jakarta in which the indoor smoking ban was not enforced (“smoking venues”), and five venues in which the ban was actively enforced (“non-smoking venues”) during Ramadan (July, 2014), and in the same venues after Ramadan (August, 2014). A purposive sampling strategy was used to select venues that represented five of the six districts of Jakarta, with consideration given to accessibility by research staff, popularity, safety, and confirmation of active cigarette smoking (a list of the venues visited is available from the authors upon request). Thus, the Thousand Islands district was excluded owing to low population and limited accessibility. Smoking venues were defined via observation of active smoking, and non-smoking venues were defined as having non-smoking signage and a zero-tolerance smoking rule, as confirmed with a pre-visit. While apparently arbitrary, this rule allowed sampling from a selection of hospitality venues, similar in size and function to smoking venues, in which the smoke-free law was actively enforced. Venues in which waterpipe smoking was observed were excluded, to reduce variability in types of SHS emission. The study protocol was considered “not human subjects research” by the Harvard T.H. Chan School of Public Health Institutional Review Board.

### 2.2. Measures

Air quality was measured using a TSI Sidepak AM510 Personal Aerosol Monitor (TSI, Inc., Shoreview, MN, USA). The Sidepak measures particulate matter using a laser-generated light scattering method. Particulate matter with a mass median aerodynamic diameter of less than 2.5 μm (PM_2.5_) was measured for minimum of 30 min indoors and for a minimum of 10 min outdoors. A calibration factor of 0.32 was used, in accordance with best practices for measurement of SHS emissions [14]. Indoor air quality was sampled from a central location within each venue, where the number of children and visitors were also counted. Outdoor measures were obtained within 10 m of the front entrance of each venue. The number of active smokers, non-smoking adults and children was measured by calculating the average of two “head counts”: The first within 15 min of commencing indoor air quality sampling, and the second during the final 15 min of sampling. Room volume (m^3^) was calculated from room dimensions measured using a Sonic Measure DM S50L (Zircon Corp., Campbell, CA, USA) Active smoker density (ASD) was calculated by dividing the mean number of active smokers by 100 m^3^. 

We collected data after *iftar* during Ramadan. In the month following Ramadan, we collected data from the same venues at the same time and day of the week as we sampled during Ramadan (although the clientele were not necessarily identical across sampling periods). Although permission was obtained from venue owners and/or managers prior to data collection, measures were collected discreetly to avoid influencing the behavior of employees or clientele. 

### 2.3. Data Transformation and Analyses 

Data were averaged across the sampling period for each venue. Average smoker density (ASD) was calculated by dividing the average number of burning cigarettes by room volume [14]. Because of non-normal distribution of most measures, PM_2.5_ data are presented with the mean and median values, and were analyzed using non-parametric analyses. Differences in means for matched pairs (e.g., inside vs. outside, and Ramadan vs. post-Ramadan) were compared using Wilcoxon matched-paired sign-rank test. Correlations were calculated using Spearman’s rho.

## 3. Results

### 3.1. Smoking Venues

**Figure 1:**
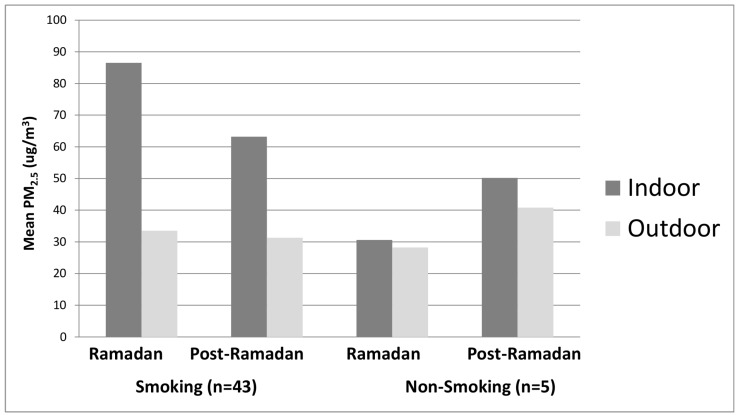
Mean PM_2.5_ levels observed in smoking and non-smoking venues during and after Ramadan.

During Ramadan, the average indoor PM_2.5_ level in smoking venues (n = 43) was 86.5 µg/m^3^ (SD = 69.5; median = 55.0; IQR = 76.0). These levels were significantly higher than the mean PM_2.5_ level of 33.5 µg/m^3^ (SD = 20.4; median = 31.0; IQR = 18.8) recorded immediately outside the same venues (*T* = 4.61; *p* < 0.001) (Figure 1). Indoor sampling occurred on average between 7:12 p.m. and 7:58 p.m., for a mean sampling duration of 46 min.

Post-Ramadan sampling of the same venues revealed a mean indoor PM_2.5_ level of 63.2 µg/m^3^ (SD = 53.2; median = 52.0; IQR = 33.3). The indoor level measured post-Ramadan was significantly higher than the mean outdoor PM_2.5_ level of 31.3 µg/m^3^ (SD = 14.8; median = 29.0; IQR = 17.8) measured at that time (*T* = 4.68; *p* < 0.001). Indoor sampling occurred on average between 7:15 p.m. and 7:54 p.m., for a mean sampling duration of 39 min.

A comparison between indoor PM_2.5_ levels measured during and post-Ramadan revealed a significantly higher indoor PM_2.5_ level during Ramadan, compared with post-Ramadan (86.5 µg/m^3^ vs. 69.5 µg/m^3^; *T* = 2.42; *p* < 0.015).

Significantly greater numbers of active smokers were observed in the smoking venues during Ramadan, compared with post-Ramadan (11.9 vs. 8.0 active smokers; *T* = 2.52; *p* = 0.012). A marginally significantly greater number of children were also observed during Ramadan (4.5 vs. 3.3 children; *T* = 1.95; *p* = 0.051). There were no differences in average smoker density (*T* = 1.33; *p* = 0.18) or the total number of non-smoking adults present (*T* = 0.97; *p* = 0.331) during Ramadan compared with post-Ramadan in smoking venues (Table 1).

A strong, positive correlation was observed between average smoker density and indoor PM_2.5_ both during Ramadan (Spearman’s rho = 0.69) and post-Ramadan (Spearman’s rho = 0.73).

### 3.2. Non-Smoking Venues

Among non-smoking venues (n = 5), there were no differences between indoor and outdoor PM_2.5_ levels measured during (30.6 µg/m^3^ vs. 28.2 µg/m^3^) or post-Ramadan (50.2 µg/m^3^ vs. 40.8 µg/m^3^), nor was there a difference between indoor PM_2.5_ levels measured during Ramadan compared with post-Ramadan (30.6 µg/m^3^ vs. 50.2 µg/m^3^) (*T*’s < 1.21, *p*’s > 0.50). It should be noted that indoor levels were insignificantly higher than outdoor levels both before and after Ramadan. Indoor sampling of non-smoking venues occurred on average during Ramadan between 8:46 p.m. and 9:23 p.m., for a mean sampling duration of 37 min, and post-Ramadan between 8:57 p.m. and 9:38 p.m., for a mean duration of 35 min.

## 4. Discussion

In Jakarta, Indonesia’s capital and most populous city, we observed a pervasive pattern of SHS contamination in venues where the indoor smoke-free law was not enforced. In those venues, PM_2.5_ levels were significantly greater than in non-smoking venues. As has been observed in Indonesia [20,21] and internationally [22,23,24], we found that non-enforcement of smoke-free laws fails to provide needed protection from SHS exposure for nonsmokers, including children. The PM_2.5_ levels observed in this study were lower than those found in a 2009 convenience sample of 17 restaurants in Jakarta (~110 µg/m^3^) [21] and in many similar venues sampled globally [16,25,26]. Nonetheless, extended exposure to the PM_2.5_ levels observed in this study would be far in excess of the annual average standard of 10 µg/m^3^ recommended by the WHO [17].

During Ramadan, an emphasis is placed on religious observance and family, culminating daily in the *iftar* gathering. SHS levels were higher in the evenings during Ramadan (when families congregated at *iftar*) than in the evenings after Ramadan. The data demonstrate that not only are smoke-free laws generally not enforced in Jakarta, but there is a high degree of cultural acceptance of smoking in the evening during Ramadan, even when children are present. This observation is consistent with a study of 15 venues in Jordan which found increased levels of SHS during the evening *iftar* celebrations, compared with earlier in the day [18]. Of particular note was the presence of children in venues without enforcement of the smoke-free law. We observed a mean point count of 4.5 children (range = 0–18) in each venue, suggesting that this number of children was present at any given time and that many more children were likely present over the course of the evening. The exposure of children to SHS in indoor spaces reflects the concerns of international public health agencies, including the WHO and the US Surgeon General, based on the association between SHS exposure and serious pediatric health outcomes [3,5].

The convergence of non-enforcement of smoke-free laws with a major cultural/religious observance—Ramadan—on SHS exposure highlights a culturally based public health problem for Indonesia. Social, cultural and economic factors may play a substantial role in shaping attitudes and behaviors that perpetuate SHS exposure in Indonesia as well as globally [27]. So, too, culturally defined gender roles may support the tacit acceptance of male smoking by nonsmoking females, as a means to maintain intra- and extra-family relationships [28,29]. A “standard” approach to tobacco control would call for renewed efforts to enforce the existing smoke-free law, together with evidence-based tobacco control measures, including appropriate health communications on risks of SHS exposure among children, as recommended by the FCTC [30]. However, enforcement might be more effective when conducted in parallel with approaches that aim to change social and cultural norms. Findings from this study, as well from other Islamic countries, suggest that indoor smoking remains highly socially acceptable even during Ramadan. Nonetheless, the pervasive religious influence that promotes the observance of Ramadan in Islamic countries could be used to channel support for the enforcement of smoke-free laws, perhaps through religious injunctions against smoking, or through existing tobacco control initiatives, including work in progress by Muhammadiyah. Engagement with religious leaders to promote smoke-free compliance in Islamic countries has so far been underutilized.

This potential approach to resolving the problem of non-adherence to indoor smoking bans in Islamic countries may have a basis in health behavioral theory. Hovell and Hughes [31] have advanced a behavioral ecological model (BEM) that explains how smoking behavior is reinforced by complex combinations of experiences and events that impact the smoker daily. According to the BEM, smoking reinforcers exist across a hierarchical array of domains, ranging from interpersonal to familial, social, cultural, and, ultimately, political influence through policies and legislation (e.g., sales tax). Discrete reinforcers, such as interpersonal approval of indoor smoking, or smoking while celebrating a religious event may combine to produce powerful synergistic influences on smoking behavior [32]. Evidence from this study suggests that cultural observance of Ramadan is closely associated with the acceptance of smoking in environments where families with children gather. The reinforcement contingency that exists between a revered cultural and religious event and indoor smoking may, over time, render smoking around and among families as highly socially normative [31]. Indeed, even broader influences may reinforce social acceptance of smoking in Indonesia. Transnational tobacco companies, including Philip Morris International and British American Tobacco, who each have a documented history of opposing smoke-free laws [33,34], have now established a presence in Indonesia [34]. Evidence suggests that these companies have sought to shape government policy by slowing the implementation of tobacco control measures [34]. With the support of the transnational tobacco industry, the Indonesian government has promulgated a 14-year “Tobacco Roadmap” (2007–2020) which has been criticized for prioritizing industry-friendly reforms to labor and tax codes before the implementation of needed tobacco control interventions [35]. Thus, the demonstration of pervasive SHS exposure in indoor venues during Ramadan also suggests that cultural and religious observances intersect with a governmental failure to enforce comprehensive smoke-free laws, perhaps with the support of transnational tobacco companies, to perpetuate indoor smoking and SHS exposure among families and children. 

The influence of religious leaders to promote abstinence from indoor smoking may disrupt the reinforcement contingencies between familial, social and cultural practices, and smoking behavior. Because noncompliance has resulted in greater exposure among children during Ramadan, religious and community leaders should establish more public requests for nonsmoking seating both inside and outside public restaurants and encourage support of the enforcement of smoke-free laws [19,32]. Such a model would require more active government enforcement of indoor smoking bans. The present findings also suggest that government regulation of tobacco industries should be enhanced. Because the tobacco industry actively promotes smoking in almost all environments [33], smoking should be completely prohibited in any public areas frequented by children. Likewise, sales should be restricted in any areas that involve children and commercial agencies (e.g., restaurants). Such changes could be both a measure of culture change and a government-supported extension of culture that does not tolerate child exposure to SHS. Such action could save millions of preventable deaths among children and their parents. The BEM could serve as a guide to such interventions and the formal analyses of the effects. 

Certain limitations should be noted. First, because a convenience sampling strategy was used, the findings cannot be taken to be representative of all venues in the greater Jakarta region. Nevertheless, a high number of venues were sampled in this study, and particular care was taken to sample venues from five major metropolitan districts, ensuring a geographically reasonable spread. Second, this study did not directly measure SHS exposure, but did so only indirectly, by monitoring PM_2.5_, a marker for SHS, in the ambient air in which children and nonsmoking adults were present. Direct measurement of SHS exposure requires analysis of a biomarker such as cotinine. Finally, it is noted that while PM_2.5_ is a common marker for SHS, it can be generated by other means, including pollution caused by cooking fuels or incense, as perhaps indicated by the PM_2.5_ levels in nonsmoking venues. It is possible that higher PM_2.5_ levels observed in smoking levels after *iftar* were attributable, at least in part, to emissions arising from cooking. By measuring outdoor PM_2.5_ levels, we were able to account for pollution due to vehicles or other outdoor pollution sources. Previous studies show that elimination of indoor smoking may decrease the indoor pollution by as much as 90%, suggesting that smoking is the main contributor of PM_2.5_ [36].

## 5. Conclusions

These findings indicate an urgent need to expand and fully enforce smoke-free laws, as recommended by the WHO’s Framework Convention on Tobacco Control [4]. While progress towards the objectives of the FCTC have been claimed by the Indonesian government [9], failure to ratify the FCTC has slowed progress toward those goals [35]. Ratification and full implementation of the FCTC by the government of Indonesia will provide a mechanism (under Article 20.4) for monitoring and responding to tobacco industry activities that undermine the implementation and enforcement of the legislation [20,37]. Evidence has shown that implementation of comprehensive bans is associated with adjustment and acceptance of bans by smokers [38], social denormalization of smoking [2], increases in voluntary home smoking bans [39,40,41], and decreased SHS exposure among children [42]. Evidence from Thailand and Malaysia suggests that regional support for comprehensive indoor bans is high, even when compliance is a challenge [43]. Community and religious organizations, local governments, and civil society should respond to the failure of current smoke-free policies in Indonesia by developing collaborative relationships in support of the enforcement of current laws [37]. With a comprehensive smoke-free policy that is properly enforced, Indonesian children and their families may benefit from smoke-free public venues not only during Ramadan, but at all times of the year.

## Figures and Tables

**Table 1:** Smoking behavior in 43 smoking venues, Jakarta 2014.

Central Tendency	Active Smoker Density		Active Smokers Present		Children Present		Non-Smoking Adults Present	
	Ramadan	Post-Ramadan	Ramadan	Post- Ramadan	Ramadan	Post- Ramadan	Ramadan	Post- Ramadan
Mean (SD)	0.21 (0.22)	0.17 (0.17)	11.9 (12.4)	8.0 (10.8)	4.5 (4.6)	3.3 (4.5)	35.6 (22.5)	30.7 (24.6)
Median (IQR)	0.13 (0.18)	0.11 (0.16)	8.0 (11.3)	6.0 (8.0)	3.0 (6.8)	2.0 (4.0)	31.0 (28.8)	22.0 (32.3)
